# Exploring the discord between pharmacy education and practice in antimicrobial stewardship

**DOI:** 10.4102/hsag.v28i0.2114

**Published:** 2023-02-27

**Authors:** Devina Chetty, Stephanie Leigh-de Rapper

**Affiliations:** 1 Department of Pharmacy and Pharmacology, Faculty of Health Sciences, University of the Witwatersrand, Johannesburg, South Africa

**Keywords:** antimicrobial stewardship, knowledge, perceptions, clinical pharmacists, pharmacy, education

## Abstract

**Background:**

Antimicrobial stewardship (AMS) is a critical global intervention aimed at optimising antimicrobial use and decreasing antimicrobial resistance (AMR) with pharmacists playing a pivotal role within AMS teams. However, AMS is not comprehensively taught in pharmacy curricula and little is known about the relevance of pharmacists’ training to meet AMS needs in South Africa.

**Aim:**

This study aimed to explore the attitudes, knowledge and perceptions of clinical pharmacists towards AMS participation and training in South Africa.

**Setting:**

This study was conducted among clinically practicing pharmacists in public and private healthcare sectors in South Africa.

**Methods:**

A quantitative exploratory research design was selected for this study. The study was conducted using a self-administered structured survey. Categorical variables were analysed using simple descriptive statistics. Mann–Whitney and Kruskal–Wallis tests were applied to determine differences between variables.

**Results:**

Pharmacists demonstrated good attitudes knowledge and perceptions towards AMS (median 4.3). There was statistical significant differences in AMS participation between pharmacists of different years of experience (*p* = 0.005), sector of employment (*p* = 0.01), position of employment (*p* = 0.015) and presence of AMS programmes (*p* = 0.004). Pharmacists indicated that their Bachelor of Pharmacy undergraduate studies inadequately prepared them for their role in AMS (median 4.3).

**Conclusion:**

Pharmacists show positive attitudes, knowledge and perceptions towards AMS. Education and training in AMS principles is obtained through master’s programmes, short courses, Continued Professional Development (CPDs) and workshops and insufficiently incorporated in undergraduate programmes.

**Contribution:**

This study confirms that undergraduate pharmacy programmes inadequately prepare pharmacists for their role in AMS.

## Introduction

Antimicrobial resistance (AMR) poses a major threat to the health of populations worldwide, puts achievements in medical advancements at risk and increases morbidity, mortality and the global economic burden (World Health Organization [WHO] 2020). In 2015, the World Health Assembly endorsed the Global Action Plan to tackle AMR with antimicrobial stewardship (AMS) as a key strategy to attain this through the promotion of the rational use of antimicrobials. Antimicrobial stewardship is a defined set of actions designed to optimise antimicrobial use for the purposes of improving clinical outcomes and patient safety. This is achieved by emphasising appropriate selection, dosage, duration of antimicrobial therapy and route of administration (Doron & Davidson 2011). In order to implement effective AMS programmes, a specialist team of healthcare professionals is needed. At an institutional level, the AMS team must be multidisciplinary consisting of a physician, clinical pharmacist, microbiologist, infection control representative and epidemiologist. There should be effective collaboration across various health sectors with pharmacist’s expertise at the centre of this (RPS 2017). Previous studies have proven the value of pharmacists and their contribution to AMS as a result of their diverse role, drug expertise and contribution within this multifaceted multidisciplinary team in hospitals (Brink et al. 2016, 2017; Van den Berg et al. 2020).

Despite the valuable and significant role pharmacists play in AMS, pharmacists face numerous barriers that impede their participation, including the lack of confidence in recommending antibiotic dose and frequency adjustments and the lack of time to participate in AMS rounds (Weier et al. 2018). Pharmacists in Australia, France, Malaysia and Qatar cited the lack of formal AMS and infectious disease education and training as an impediment to their involvement in AMS activities (Khan et al. 2016; Nasar et al. 2019; Rizvi et al. 2019; Weier et al. 2018). Amidst the global health crisis of AMR, the acute respiratory syndrome coronavirus (SARS-CoV2) resulted in the outbreak of the Coronavirus Disease (COVID-19) pandemic in 2019 creating unprecedented challenges to health systems globally (Weiner-Lasting et al. 2021). The COVID-19 pandemic overwhelmed healthcare systems; clinical teams focused attention and resources on managing the pandemic, disrupting established services as well as AMS activity (Ashiru-Oredop et al. 2021). Antimicrobial use increased, exceeding the incidence of bacterial coinfections and secondary infections, suggesting inappropriate use and raising concerns about the impact on AMR (Grau et al. 2021).

Education, training and development of pharmacists and other disciplines forming part of the AMS team are crucial to achieving AMS objectives and reducing AMR (WHO 2015). The WHO published a comprehensive guideline for health educators on how to develop learning content of AMR with the ultimate goal of equipping healthcare workers with the skills and competency to manage antimicrobials according to their scope of practice (WHO 2019). The South African National Department of Health recommends the inclusion of AMS in pharmacy undergraduate curricula; however, training of pharmacists in infectious diseases and AMS is not yet formalised (Schellack et al. 2018). Not all universities in South Africa, who offer pharmacy training, offer a postgraduate clinical pharmacy programme. Despite some availability of formal training, there is a lack of acknowledgment of the role and scope of practice of clinical pharmacists within South African Pharmacy Council registration (Bronkhorst, Schellack & Gous 2020). Pharmacists with a postgraduate or undergraduate qualification are both registered as a pharmacist with differentiation only in the sector of practice (Gray, Riddin & Jugathpal 2016). Pharmacists with a postgraduate qualification in clinical pharmacy should be registered as a specialist pharmacist with clearly defined roles and responsibilities (Bronkhorst et al. 2020). This lack of differentiation leads to limited focus in undergraduate training requiring pharmacists to be trained in a master’s degree to fill this gap; this too is not regulated in scope and structure of learning. The lack of differentiation in scope of practice between a graduate with a Bachelor of Pharmacy (B.Pharm) qualification and one with a Master of Pharmacy (M.Pharm) qualification has resulted in pharmacists with either pharmacy qualification performing clinical functions (Bronkhorst et al. 2020) and involved in AMS activities (Khan et al. 2020).

In South Africa, little is known about the relevance of pharmacists’ training to meet AMS needs in South Africa and little is known about clinically practicing South African pharmacists’ attitude, knowledge and perceptions on AMS and pharmacy education. Therefore, this study aimed to explore the attitudes, knowledge and perceptions of clinical pharmacists towards AMS participation and training in South Africa.

## Research methods and design

### Study design and sites

A quantitative exploratory cross-sectional research design was selected for this study. The study population consisted of pharmacists employed as clinical pharmacists as well as pharmacists performing clinical functions and involved in AMS in both the public and private hospitals in South Africa.

### Sample size

Purposive sampling was undertaken for this study and the study sample size was determined based on the response rate of questionnaires. Response rates are approximately 25% – 30% indicating a minimum target amount of 20 returned questionnaires. The South African Society of Clinical Pharmacy (SASOCP) was approached to distribute the survey to its members. The SASOCP is a society formed in response to the resurgent interest in clinical pharmacy with 85 paid members at the time the survey was distributed. A sample size of 55 was obtained, yielding a response rate of 64.7%. Out of a maximum of 55 responses, not all respondents answered and rated each variable from questions two to nine. Hence, the number of responses received for each variable is reflected by a change in *n*-value. The percentage is then calculated based on the number of responses received for that variable.

### Data collection and instruments

A self-administered structured survey was designed specifically for this study, incorporating questions from previous instrument designs of similar studies (Burger et al. 2016; Khan et al. 2020; Nasar et al. 2019; Weier et al. 2018). The questionnaire was divided into three sections, including (1) participants’ demographics, (2) questions relating to participants’ perceptions and knowledge towards AMS, participation in AMS and factors affecting participation in AMS and (3) questions relating to participant acquisition of knowledge on AMS. The self-administered survey was emailed to SASOCP’s executive committee. A member of the executive committee emailed the survey to SASOCP members.

### Data analysis

Data were analysed by using StataSE 17. Categorical variables such as demographics, attitudes and perceptions of participants were analysed using simple descriptive statistics measuring numbers, frequency and percentages. Normality of data was assessed using Kolmogorov–Smirnov and Shapiro–Wilks tests. Mann–Whitney and Kruskal–Wallis tests were applied to determine differences in perceptions, knowledge and participation of pharmacists. Both tests were also used to check differences among gender, age, years of experience, sector of employment, current position of employment and groups. A *p*-value of less than 0.05 was considered statistically significant. Reliability coefficient was tested, Cronbach’s alpha score of 0.90 for perceptions, 0.91 for participation and 0.91 for factors affecting participation.

### Ethical considerations

Ethics approval was obtained from Human Research Ethics Committee (Medical) of the University of the Witwatersrand, ethics clearance number M210937. A study information sheet explained the study in detail and the benefits and risks of participating in the study. Completion of the questionnaire was anonymous and data were confidentially maintained.

## Results

### Participants’ demographics

Table 1 represents the data derived for participants’ demographics. Female participants represented 72.73% (*n* = 40) of the study population and male participants represented 27.27% (*n* = 15). The majority of the participants were between the ages of 30 and 39 years 45.45% (*n* = 25), have an M. Pharm qualification 65.46% (*n* = 36) with more than 10 years’ experience 50.91% (*n* = 28). Pharmacists in the private sector represented 81.82% (*n* = 45) of the responses. The majority of participants were employed as pharmacists 32.73% (*n* = 18), followed by 29.09% (*n* = 16) clinical pharmacists and 27.27% (*n* = 15) ward pharmacists. Forty-one participants (74.55 %) indicated there is an AMS programme at their institution of employment while 25.45% (*n* = 14) indicated they work without an AMS programme.

**TABLE 1:** Demographics of participants (*n* = 55).

Variable	Category	Frequency	%
Gender	Male	15	27.27
	Female	40	72.73
Age (years)	20–29	13	23.64
	30–39	25	45.45
	40–49	9	16.36
	50–59	6	10.91
	60	2	3.64
Qualification	Dip. Pharm	0	0
	B. Pharm	13	23.65
	M. Pharm	36	65.46
	PhD	6	10.91
Years of experience	< 1 year	2	3.64
	1–4 years	13	23.64
	5–9 years	12	21.82
	10 years and more	28	50.91
Sector of employment	Private	45	81.82
	Public	10	18.18
Current position	Pharmacist	18	32.73
	Ward Pharmacist	15	27.27
	Senior Pharmacist	1	1.82
	Assistant Pharmacy Manager	1	1.82
	Pharmacy Manager	4	7.27
	Clinical Pharmacist	16	29.09
AMS programme at institution of employment	Yes	41	74.55
	No	14	25.45

AMS, antimicrobial stewardship.

Dip.Pharm, Diploma in Pharmacy; B.Pharm, Bachelor of Pharmacy; M.Pharm, Master of Pharmacy; PhD, Doctor of Philosophy.

### Exploring the attitudes and knowledge of clinical pharmacists’ towards antimicrobial stewardship

Table 2 displays data derived from participants responses to perception and knowledge towards AMS. Of a maximum score of 5 (100%) for knowledge and perceptions, participants obtained a median score of 4.3 (IQR 4.6–4.0) indicating consistent good knowledge and perception towards AMS. The majority of participants (96.22%, *n* = 51) agreed and/or strongly agreed on the definition of AMS, median 5 (IQR 5–5). A total of 47 (88.68%) agreed/strongly agreed that an AMS team should consist of a multidisciplinary team including a physician, infectious disease specialist, pharmacist and a microbiologist. Forty-five (84.88%) participants disagreed and/or strongly disagreed that a goal of AMS is to increase the use of antimicrobials while 39 participants (73.58%) agreed and/or strongly agreed that a goal of AMS is to reduce hospital stay. Majority of participants (*n* = 50, 94.33%) agreed and/or strongly agreed that optimising antimicrobial use and reducing antimicrobial resistance is a goal of AMS. Participants unanimously agreed and/or strongly agreed that pharmacists play a critical role in AMS to promote optimal use of antimicrobials (median 5; IQR 5–5), educate healthcare professionals (median 5; IQR 5–4), lead and support AMS activities (median 5; IQR 5–4) and to recommend appropriate therapy or de-escalate (median 5; IQR 5–4). Table 3 shows two-sample Wilcoxon rank-sum (Mann–Whitney) and Kruskal–Wallis equality-of-populations rank that was used to test statistical differences in median total perception, total participation by demographic characteristic. No statistical difference was found.

**TABLE 2:** Participants’ perception and knowledge towards antimicrobial stewardship and participants’ median scores.

Variable	Responses (%)										Median	IQR
	SD		D		N		A		SA			
	*n*	%	*n*	%	*n*	%	*n*	%	*n*	%		
AMS is a set of coordinated initiatives to promote the appropriate and judicious use of antimicrobials (*n* = 53)	1	1.89	0	0.0	1	1.89	11	20.75	**40**	**75.47**	5	5–5
AMS team should consist of a multidisciplinary team including a physician, infectious disease specialist, pharmacist and a microbiologist (*n* = 53)	1	1.89	4	7.55	1	1.89	12	22.64	**35**	**66.04**	5	5–4
The goal of AMS is to increase the use of antimicrobials (*n* = 53)	**40**	**75.45**	5	9.43	0	0.0	1	1.89	7	13.21	5	5–5
The goal of AMS is to reduce hospital stay (*n* = 53)	1	1.89	5	9.43	8	15.09	15	28.30	**24**	**45.28**	4	5–3
The goal of AMS is to decrease duration of therapy and ensure therapeutic efficacy (*n* = 53)	1	1.89	1	1.89	4	7.55	11	20.75	**36**	**67.92**	5	5–4
The goal of AMS is optimise antimicrobial use (*n* = 54)	1	1.89	0	0.0	3	3.77	4	7.55	**46**	**86.79**	5	5–5
The goal of AMS is to reduce antimicrobial resistance (*n* = 53)	1	1.89	1	1.89	1	1.89	11	20.75	**39**	**73.58**	5	5–4
Pharmacists play a critical role in AMS to promote optimal use of antimicrobials (*n* = 53)	2	3.77	0	0.0	1	1.89	7	13.21	**43**	**81.13**	5	5–5
Pharmacists play a critical role in AMS to educate healthcare professionals (*n* = 53)	1	1.89	0	0	1	1.89	14	26.42	**37**	**69.81**	5	5–4
Pharmacists play a critical role in AMS to lead and support AMS activities (*n* = 53)	1	1.89	0	0	2	3.77	15	28.30	**35**	**66.04**	5	5–4
Pharmacists play a critical role in AMS to recommend appropriate therapy or de-escalate (*n* = 53)	1	1.89	0	0	1	1.89	14	26.42	**37**	**69.81**	5	5–4

**Total perception score**											**4.3**	**4.6–4.04**

*Source:* Adapted from Burger, M., Fourie, J., Loots, D., Mnisi, T., Schellack, N., Bezuidenhout, S. et al., 2016, ‘Knowledge and perceptions of antimicrobial stewardship concepts among final year students in pharmacy schools in South Africa’, Southern African Journal of Infectious Diseases 31(3), 84–90. https://doi.org/10.4102/sajid.v31i3.83

SD, strongly disagree; D, disagree; N, neutral; A, agree; SA, strongly agree; AMS, antimicrobial stewardship; IQR, interquartile range.

Correct answers are indicated in bold font.

**TABLE 3:** Variation in participants’ perception, knowledge and practices concerning antimicrobial stewardship by characteristic.

Variable	Category	Total perception			Total participation		
		Median	Rank	*p*	Median	Rank	*p*
Gender†	Male	4.36	426.00	0.909	7	368.50	0.330
	Female	4.45	1114.00		7.95	1171.50	
Age‡	20–29	4.27	380.50	0.800	6.3	315.50	0.212
	30–39	4.45	710.00		7.7	688.0	
	40–49	4.27	228.00		8.3	355.00	
	50–59	4,22	144.00		7.5	144.50	
	60	4.63	77.50		7.8	57.00	
Qualification‡	Dip. Pharm	0	0.00	0.389	0	0.00	0.422
	B. Pharm	4.27	333.50		6.4	308.00	
	M. Pharm	4.45	1078.50		7.8	1030.50	
	PhD	4.09	128.00		8.3	201.50	
Years of experience‡	< 1 year	4.36	93.00	0.087	9.8	106.00	0.005
	1-4 years	4.18	263.00		6.2	216.00	
	5-9 years	4.5	263.00		7.4	343.00	
	10 years and more	4.4	855.50		8	875.00	
Sector of employment†	Private	4.45	1325.50	0.149	8	1377.50	0.010
	Public	4.13	214.50		5.9	162.50	
Current Position‡	Pharmacist	4.45	522.50	0.975	5.4	342.50	0.015
	Ward Pharmacist	4.45	439.50		8.3	541.50	
	Senior Pharmacist	4.45	31.00		9.2	48.00	
	Assistant Pharmacy Manager	4.18	17.50		8.9	45.00	
	Pharmacy Manager	4.31	105.50		6.2	73.50	
	Clinical Pharmacist	4.31	424.00		8	489.50	
AMS programme at institution of employment†	Yes	4.27	1124.50	0.647	8	1294.50	0.004
	No	4.5	415.50		5.2	245.50	

AMS, antimicrobial stewardship; Dip.Pharm, Diploma in Pharmacy; B.Pharm, Bachelor of Pharmacy; M.Pharm, Master of Pharmacy; PhD, Doctor of Philosophy.

† , Two-sample Wilcoxon rank-sum (Mann–Whitney) test.

‡ , Kruskal–Wallis equality-of-populations rank test.

### Exploring the perceptions of clinical pharmacists’ towards antimicrobial stewardship participation and the factors affecting participation

Table 4 displays data derived from participant’s responses to participation in AMS activities and variation in the levels of participation among the different positions of employment. Of a maximum score of 10, respondents obtained a total median score of 7.8 (IQR 8.5–6.0) demonstrating that respondents had very good participation in AMS activities. Participants almost always monitored AMS duration (median 9; IQR 10–7), consumption (median 9; IQR 10–7) and participated in AMS ward rounds (median 9; IQR 10–5). Participants mostly communicated with prescribers if unsure about antibiotic appropriateness (median 8; IQR 10–7), felt confident when communicating with prescribers (median 8; IQR 9–6) and sought additional information before deciding to dispense an antibiotic (median 8; IQR 9–5). Majority of participants indicated they take part in AMS campaigns to promote rational use of antimicrobials (median 8; IQR 10–5). To a lesser extent, participants screen antibiotic scripts in accordance with local guidelines (median 7; IQR 10–5), collaborate with other health professionals if needed, before dispensing an AMS prescription (median 7; IQR 8–5) and educate patients on appropriate use and resistance-related issues (median 7; IQR 9–5). The differences between total overall participation and demographic groups were analysed; the results are displayed in Table 3. No statistical differences were found between gender, age and qualification (*p* = 0.33; 0.212 and 0.422, respectively). The various current positions of employment showed statistical differences *p* = 0.015 with ward pharmacists, senior pharmacists, assistant pharmacy managers and clinical pharmacists showing higher levels of participation compared with pharmacists (median 5.4) and pharmacy managers (median 6.2). Participants employed as pharmacists show low levels of participation in monitoring AMS duration (*p* = 0.015); monitoring antimicrobial consumption (*p* = 0.13); AMS rounds (*p* = 0.006); participating in AMS campaigns (*p* = 0.03) and screening AMS prescriptions in accordance with local guidelines before dispensing, (*p* = 0.006), medians = 5 compared with clinical pharmacists, ward pharmacists and senior pharmacists. Pharmacists with less than a year of experience showed higher levels of participation (median 9.8; *p* = 0.005) followed by pharmacists with more than 10 years of experience (median 8; *p* = 0.005). Pharmacists employed in private sector showed higher participation (median 8; *p* = 0.01) compared with those employed in the public sector. Pharmacists working in an institution with an AMS programme demonstrated much higher levels of participation in AMS (median 8) compared with those working without an AMS programme (median 5.2), *p* = 0.004. Supplementary Table 1 shows data derived from factors limiting participation. Respondents identified the following factors as factors that greatly impact participation: number of pharmacists, a lack of support from hospital administration, poor communication between prescriber and pharmacist, pharmacist’s recommendation not seen as credible, prescribers not willing to engage with pharmacists and doctors not caring about antibiotic prescribing or restrictions (median = 9). Time spent on other activities, poorly established AMS committee, limited knowledge on appropriate AMS utilisation, the lack of confidence and the lack of education and training in AMS showed significant impact on participation (median = 8). The majority of participants (*n* = 11; 22%) rated ‘participation won’t make a difference’ as a factor that somewhat limits participation.

**TABLE 4:** Participation in antimicrobial stewardship and variation in participation among different positions of employment.

Variable	Median	IQR	Variation among different positions of employment												
			Pharmacist		Ward Pharm		Senior Pharm		Assistant PM		PM		Clinical Pharm		*p*
			Median	Rank	Median	Rank	Median	Rank	Median	Rank	Median	Rank	Median	Rank	
I screen antibiotic prescriptions in accordance with local AMS guidelines before dispensing	7	10–5	5	323.50	8	533.50	10	46.00	10	46.00	5	52.50	7	431.50	0.006
I collaborate with other healthcare professionals if need be before I dispense an AMS prescription	7	8–5	5	330.00	8	498.00	8	38.00	10	48.50	5	48.00	7	468.50	0.013
I communicate with the prescriber if I am unsure about the appropriateness of an antibiotic prescription	8	10–7	7	372.50	8	453.50	8	25.50	8	25.50	7	79.00	9	475.00	0.407
I am confident when I communicate with prescribers regarding and AMS prescription	8	9–6	8	442.00	8	447.50	9	38.00	8	30.00	6.8	73.50	8	400.00	0.783
I sought additional clinical information (drug interactions; ADRS, allergies) before deciding to dispense the Antibiotic prescribed	8	9–5	8	405.00	8	415.50	10	47.00	8	29.00	5	70.00	8	464.50	0.397
I take part in AMS campaigns to promote rational use of antimicrobials	8	10–5	5	336.00	9	495.50	10	45.00	8	25.50	6	58.00	8	471.00	0.036
I educate patients on appropriate use and resistance related issues	7	9–5	7	466.50	7	438.00	8	33.00	7	28.00	7	118.00	6	347.50	0.907
I monitor antimicrobial duration	9	10–7	5	287.50	10	516.00	10	42.50	10	42.50	8.5	106.00	9	436.50	0.015
I monitor antimicrobial consumption	9	10–7	5	277.50	10	519.50	9	28.50	10	43.50	8.5	110.00	9	452.00	0.013
I participate in AMS rounds	9	10–5	5	273.00	10	509.50	10	44.00	10	44.00	5.5	91.50	9	469.00	0.006

**Total participation score**	**7.8 (8.5–6.0)**														

Source: Adapted from Weier, N., Tebano, G., Thilly, N., Demoré, B., Pulcini, C. & Zaidi, S.T.R., 2018, ‘Pharmacist participation in antimicrobial stweardship in Australian and French hospitals: A cross sectional nationa wide survey’, Journal of Chemotherapy 73(3), 804–813. https://doi.org/10.1093/jac/dkx435 and Nasar, Z., Higazy, A. & Wilbur, K., 2019, ‘Exploring the gaps between education and pharmacy practice on antimicrobial stewardship: A qualitative study among pharmacists in Qatar’, Advances in Medical Education and Practice 10, 287–295. https://doi.org/10.2147/amep.s198343

Assistant PM, assistant pharmacy manager; Pharm, pharmacist; PM, pharmacy manager; AMS, antimicrobial stewardship.

Participants were asked to rate their level of participation in the following AMS activities where 0 = never; 5 = occasionally and 10 = always.

### Exploring the attitudes and perception of clinical pharmacists’ on the relevance of pharmacy training on pharmacy practice with regard to antimicrobial stewardship

Table 5 shows data from participant’s perceptions on the relevance of pharmacy training on pharmacy practice with regard to AMS. Participants indicated that they are most likely to acquire knowledge on AMS at conferences, workshops or other educational activities (often/always *n* = 40; 80%) and least likely to acquire this knowledge in undergraduate programmes (often/always *n* = 14; 28%). Table 6 displays data derived from participant rating of the extent to which their undergraduate studies and or postgraduate studies prepared them for their role in AMS. Participants indicated that undergraduate studies inadequately prepared them for their role in AMS (median 4.5) whereas M. Pharm studies adequately prepared them for their role in AMS (median 8). Table 7 shows data derived from participant ratings of where they think a comprehensive course in AMS and infectious disease would be most beneficial. Participants unanimously agreed that a comprehensive course in AMS and infectious disease would be most beneficial. in undergraduate programmes (agree/strongly agree *n* = 48; 100%).

**TABLE 5:** Where are you most likely to acquire knowledge on antimicrobial stewardship.

Variable	Responses (%)									
	Never		Rarely		Occasional		Often		Always	
	*n*	%	*n*	%	*n*	%	*n*	%	*n*	%
Undergraduate programme (*n* = 50)	7	14.00	11	22.00	18	36.00	8	16.00	6	12.00
Master’s programme (*n* = 49)	4	8.16	2	4.08	7	14.29	17	34.69	19	38.78
Short Courses initiated by self (*n* = 50)	0	0.00	2	4.00	12	24.00	22	44.00	14	28.00
Short courses initiated by employer (*n* = 49)	5	10.20	9	18.37	16	32.65	15	30.61	4	8.16
CPD activities (*n* = 50)	0	0.00	7	14.00	12	24.00	26	52.00	5	10.00
Conferences, workshops or other educational activities are required (*n* = 50)	1	2.00	5	10.00	4	8.00	21	42.00	19	38.00

CPD, Continued Professional Development.

**TABLE 6:** How did your studies during university prepare you to for this role?

Variable	Responses											Median	IQR
	0	1	2	3	4	5	6	7	8	9	10		
Undergraduate B. Pharm studies (*n* = 48)	10 (20.83)	6 (12.50)	1 (2.08)	4 (8.33)	3 (6.25)	10 (20.83)	2 (4.17)	5 (10.42)	5 (10.42)	1 (2.08)	1 (2.08)	4.5	5.5
Postgraduate M. Pharm studies (*n* = 43)	3 (6.98)	0 (0.00)	0 (0.00)	1 (2.33)	0 (0.00)	7 (16.28)	1 (2.33)	6 (13.95)	7 (16.28)	3 (6.98)	15 (34.88)	8	5

IQR, interquartile range; B.Pharm, Bachelor of Pharmacy; M.Pharm, Master of Pharmacy.

Participants were asked to rate how university teaching prepared them for their role in antimicrobial stewardship activities where 0 = never; 5 = occasionally and 10 = always.

**TABLE 7:** Where do you think a comprehensive course in antimicrobial stewardship and infectious diseases would be most beneficial.

Variable	Responses (%)									
	SD		D		N		A		SA	
	*n*	%	*n*	%	*n*	%	*n*	%	*n*	%
Undergraduate programme (*n* = 48)	0	0.00	0	0.00	0	0.00	25	52.08	23	47.92
Master’s programme (*n* = 48)	2	4.17	1	2.08	1	2.08	15	31.25	29	60.42
CPDs (*n* = 48)	0	0.00	2	4.17	2	4.17	28	58.33	16	33.33
Short courses (*n* = 48)	0	0.00	1	2.08	2	4.17	22	52.08	23	47.92

SD, strongly disagree; D, disagree; N, neutral; A, agree; SA, strongly agree; CPD, Continued Professional Development.

Supplementary Table 2 shows data derived from participants’ rating the importance of AMS topics as low, moderate or high in importance. All participants agreed that dose optimisation is highly important (*n* = 48; 100%). Followed by infectious disease (*n* = 43; 89.58%), patient individualisation and surgical prophylaxis (*n* = 39; 81.25%). All participants viewed agent of choice as moderately important (*n* = 48; 100%). Only 28 participants (58.33%) rated standard treatment guideline compliance and team dynamics as a highly important, and 25 participants (52.08%) rated standard treatment guideline compliance as highly important. Supplementary Table 3 shows data derived from rating teaching approaches for improving AMS. All teaching approaches were rated on the upper end of the scale towards highly rated. Participants scored integrated case base and problem-based learning as a median of nine. Participants scored the remaining three approaches: didactic lectures, early clinical internship and small group class assignments and case study analysis. All teaching approaches were rated on the upper end of the scale towards a maximum score of 10 indicating that they were highly rated.

## Discussion

Pharmacists have a responsibility to take a prominent role in AMS programmes at institutional level. Previous studies found that a lack of education and training are key barriers to pharmacists’ participation in AMS programmes and activities (Nasar et al. 2019; Weier et al. 2018). To the best of the authors’knowledge, this is the first study to explore the attitudes, knowledge and perceptions of clinical pharmacists’ on AMS participation and the discord between pharmacy education and actual clinical practice in South Africa.

Numerous studies have shown the growing awareness, positive perceptions and good knowledge of pharmacists towards AMS (Erku 2020; Khan et al. 2016; Saleh et al. 2020; Sarwar et al. 2018; Tang et al. 2020). This study shows that South African pharmacists employed as clinical pharmacists as well as pharmacists performing clinical functions and involvement in AMS in both the public and private hospitals had equally good attitudes, perceptions and knowledge towards AMS. There was overwhelming evidence that participants identified with the critical role pharmacists play in all facets of AMS. Similarly, a study by Saleh et al. (2020) showed that public sector hospital pharmacists agreed that pharmacists have a responsibility to take a prominent role in AMS. These findings are encouraging as positive perceptions of pharmacists reinforce the concept of developing awareness of AMS programmes (Khan et al. 2016) and enhance adherence to AMS guidelines (Kalungia et al. 2019).

The SASOCP position statement on the pharmacists’ role in AMS 2018 defines the antibiotic stewardship pharmacists’ role, which includes the preservation of antibiotics through monitoring, evaluation and guidance of appropriate antibiotic use (Schellack et al. 2018). Hence, it is reassuring that the result of this study confirms that South African pharmacists focused their attention on monitoring antimicrobial duration and consumption and participated in AMS ward rounds. The findings of this study strengthen the preceding work confirming the growing involvement and value pharmacists in AMS participation (Kalungia et al. 2019; Saleh et al. 2020; Tang et al. 2020).

Although pharmacists’ overall participation in AMS activities was good, it is important to take note of the varying degrees of participation within the demographic groups with statistical significant differences. Pharmacists in private sector reported higher levels of participation in AMS activities compared with pharmacists in the public sector. This is most likely because of the overwhelming presence of an AMS programme in the private sector (92.68%) compared with only 7.32% in the public sector. Well-established AMS programmes offer a structured and guided approach to AMS activities (Majumder et al. 2020). The support and collaboration offered among a multidisciplinary AMS team also enhances a pharmacists participation in AMS activities (Weier et al. 2018). Studies reveal that the private sector shows a more co-ordinated implementation of AMS activities across hospital groups whereas the public sector shows successful implementation of AMS activities across a few hospitals (Chetty et al. 2019). The main reasons for poor implementation of AMS activities in public sector were a lack of stewardship resources, Information Technology (IT) microbiology and infectious disease specialists support (Chetty 2021; Engler et al. 2020).

Job-specific profiles with clearly defined roles and responsibilities vary among the different positions of employment. Ward and clinical pharmacists have a more focused role in AMS activities and are responsible for delivering on key performance indicators in clinical pharmacy processes, among other clinical pharmacy functions; however, AMS activities should not only be limited to AMS team members but also include dispensary pharmacists (Schellack et al. 2018). Therefore, it is encouraging to observe that participants in this study who were employed as pharmacists show satisfactory participation in AMS activities albiet to a lesser extent compared with ward and clinical pharmacists.

Despite good attitudes, knowledge, perception and participation in AMS, pharmacists indicated that various prescriber, organisational and educational factors may significantly limit their levels of participation. Prescriber factors (poor communication, pharmacists recommendation not creditable/not well received by doctors, prescribers not willing to engage with pharmacists, and doctors not caring about antibiotic prescribing or restriction) were highly rated factor limiting pharmacists’ participation. This could be because of doctors lack of knowledge and understanding about pharmacists’ roles, competencies and activities (Bechet et al. 2016). Mutual appreciation, trust and respect of each health professionals’ competence are factors that can foster effective collaboration between pharmacists and doctors (Löffler et al. 2017). Pharmacists need to invest sufficient time during ward rounds to create more face-to-face opportunities to build and enhance professional relationships with prescribers. However, organisational factors of limited time and resources allocated to AMS activities have been cited as a factor impeding pharmacists participation in AMS (Ji et al. 2021; Kalungia et al. 2019; Saleh et al. 2020; Tang et al. 2020). Likewise, pharmacists of this study rated similar organisational factors; number of pharmacists, a lack of support by hospital administration, time spent on other activities (paper-work, dispensing and stock management), and a poorly established AMS committee as factors impeding their participation. Evidence has shown that dedicated and sustained funding and hospital leadership support are predictors for an effective Antimicrobial Stewardship Programme (ASP) (Abubakar & Tangiisuran 2020). Hospital leadership and AMS committees in South Africa should review the core elements of hospital the ASP as defined by the Centres for disease control and prevention (CDC), leadership committement should be enhanced to dedicate the necessary human and financial capital to AMS programmes. Pharmacists reports of inadequate time to participate in AMS activities because of other daily tasks such as dispensing and stock management imply that human and financial capital is insufficiently distributed within pharmacy departments to enable sustained work on AMS activities. Re-enforcing core elements of a ASP, hospital leadership and accountability (e.g. identifying a single leader responsible for programme outcomes) can inspire clinicians to play a crucial role on AMS (Brink 2015). The other relevant core element is education; health professionals should be educated about disease state management, antimicrobial resistance and optimal prescribing. There is scope within this core element to educate doctors of the roles, responsibilities and core competence of the AMS committee members. This form of interprofessional education improves the health care professionals’ understanding of each others role and responsibilities, thus significantly improving interprofessional knowledge and attitudes towards collaborative AMS (MacDougall et al. 2017).

The importance of incorporating AMS training within undergraduate healthcare programmes has been emphasised by the WHO, Global Action on Antimicrobial Resistance (WHO 2015).

Universities worldwide have heeded this call and incorporated AMS training in undergraduate and postgraduate healthcare programmes; however, there is variation in the extent to which AMS training is included, as well as differences in pedagogies (Abubakar & Tangiisuran 2020; Ji et al. 2021; Kalungia et al. 2019; Saleh et al. 2020; Weier et al. 2018). In studies where there was some inclusion of AMS concepts in undergraduate programmes, there were reports of misalignment in the form of irrelevant syllabus to AMR, no courses on AMS practice (Khan et al. 2021), difference in elements of stewardship (Castro-Sánchez et al. 2016), and inconsistent application of content by different pharmacy schools in the same country (Justo et al. 2014). A study by Burger et al. (2016) showed a similar finding among pharmacy schools in South Africa. This study showed that eight universities across South Africa included some form of AMS education; however, there were significant differences in AMS education between the different universities (*P* < 0.001).

The study by Burger et al. (2016) further reported significant differences in AMS knowledge between students from the various universities. The results of this study support the findings of the aforementioned study by Burger et al. (2016). The participants of this study indicated that they acquire most of their AMS knowledge from conferences, workshops, masters programmes and short courses. Although only 14% of participants indicated they acquired AMS knowledge from undergraduate programmes, this is an indication that undergraduate pharmacy programmes in South Africa include some form of AMS training. This is expected as the pharmacy curriculum is aimed at developing a generalist in pharmacy and not a specialist, requiring continued growth and development of the pharmacist after graduation to meet the needs of clinical practice. The results of this study also show that pharmacists with less than a year of experience have higher levels of participation in AMS activities compared with pharmacists with more than 10 years of experience; this further substantiates the positive influence by the undergraduate curriculum.

Pharmacy schools should note that pharmacists were all in agreement that a comprehensive course in AMS and infectious disease would be most beneficial if taught in undergraduate programmes. Highly rated topics for inclusion in AMS curricula were infectious disease, dose optimisation, patient individualisation and surgical prophylaxis. The agent of choice was rated as moderately important. In order to be effective members of the AMS team pharmacists require skills and knowledge in antimicrobial use, infectious disease as well as leadership skills to implement AMS programmes and work effectively within a multidisciplinary team (Weier et al. 2018). Majority of pharmacists rated interpersonal and communication skills development and team dynamics as a topic of high importance. Pharmacists rated all teaching approaches including; didactic lectures, integrated case-based learning, problem-based learning, early internship and small group assignments, as highly beneficial in improving undergraduate AMS coverage. Internships offer the opportunity for practical experiential learning, which helps provide a holistic view and application of knowledge and skills (Teoh et al. 2020).

Although it is important to create a solid foundation of AMS knowledge at undergraduate level, AMS education and training should also continue throughout health professional careers to shape and reaffirm their attitudes and behaviours to AMS (Majumder et al. 2020). The importance of continuous professional development to keep abreast with the latest antimicrobial practices and recommendation is highlighted in a study by Teoh in 2020. The AMS education should be available as continuing medical education, which provides evidence-based information in the form of lectures, tutorials and grand rounds (Majumder et al. 2020).

The findings of this study suggest that clinical pharmacists, pharmacists involved in clinical work, and AMS in South Africa have good attitudes, knowledge and perceptions towards AMS and have good levels of participation in AMS activities. Despite the presence of AMS programmes, pharmacist indicated that many factors could limit their level of participation in AMS activities suggesting the need to enhance and develop AMS programmes. Furthermore, the study’s findings confirm that AMS principles are insufficiently incorporated in undergraduate pharmacy programmes. However, informal education and training in the form of short courses, CPD activities, conferences and workshops are available, which pharmacists utilise to improve their knowledge on AMS.

## Limitations

There are a few limitations to this study. Firstly, the study was conducted among SASOCP members, clinical pharmacists and pharmacists involved in clinical work and AMS who are not SASOCP members did not get an opportunity to participate in this study. Secondly, there was only a small representation from the public sector. The results from the public sector may differ with greater representation.

## Implications and recommendations

As this study targeted SASOCP members and had minimum representation from the public sector, further large-scale studies are warranted to validate these results by including a greater number of pharmacists. This study highlighted that pharmacy schools in South Africa incorporate AMS training into their undergraduate curriculum; it is therefore recommended that a study be carried out to determine what pharmacy schools are teaching with regard to AMS. The findings from this study support the recommendation to pharmacy schools to implement systematic education and training for pharmacists, starting from instilling basic principles of AMR, AMS, infectious disease and interpersonal skill development in undergraduate programmes, to continuous education and development in the form of short courses and CPD. The Department of Health should redirect resources to improve AMS programmes in public sectors. Hospital administrators in private and public sectors are encouraged to monitor and support AMS programmes within their institutions. There is strong evidence that doctors are unwilling to work with clinical pharmacists; hence, a study should be conducted to evaluate the factors that influence interprofessional collaboration between doctors and clinical pharmacists.

## Conclusion

This study found that clinical pharmacists, pharmacists involved in clinical work and AMS in South Africa have good attitudes, knowledge and perception towards AMS. Pharmacists of all positions of employment participate in AMS with pharmacists employed as clinical and ward pharmacist’s showing greater levels of participation. Masters programmes, short courses, CPDs, conferences and workshops are avenues available for pharmacists to acquire AMS knowledge; however, pharmacists are of the view that an undergraduate AMS programme will be most beneficial to bridge the current gap between education and practice.
